# Deciphering Pesticide Stress Responses in Rice Through Integrated Multi-Omic Assessment

**DOI:** 10.3390/toxics14030210

**Published:** 2026-02-28

**Authors:** Azam Safarnejad, Joaquim Jaumot, Stefan Platikanov

**Affiliations:** Department of Environmental Chemistry, Institute of Environmental Assessment and Water Research (IDAEA), Spanish National Research Council (CSIC), Jordi Girona 18-26, E008034 Barcelona, Spain; azam.safarnejad@idaea.csic.es (A.S.); stefan.platikanov@idaea.csic.es (S.P.)

**Keywords:** rice (*O. sativa*), pesticide stress, phytotoxicity, multi-omics, transcriptomics, proteomics, metabolomics, crop resilience

## Abstract

Pesticides are widely used in rice cultivation for pest control to guarantee crop productivity. Intensive use of these chemicals causes harmful effects on rice plants, such as physiological and biochemical stress responses. Such stress is often expressed as oxidative damage, disruption of metabolic balance, and a reduction in plant resilience to environmental challenges. In recent years, omic technologies (such as transcriptomics, epigenomics, proteomics, and metabolomics) have contributed to identifying molecular pathways affected by pesticide exposure. However, no comprehensive synthesis of rice-specific omic evidence currently exists, limiting translational applications. These omic studies revealed activation of detoxification-related enzymes and transporters, alongside changes in antioxidant defenses, hormone-mediated signaling, and membrane remodeling. This review presents current omic-based approaches used to investigate pesticide-induced stress in rice. It focuses on molecular responses including changes in gene expression, enzymatic detoxification, metabolic reprogramming, and stress signaling pathways. The review also highlights how multi-omic integration can contribute to a more holistic understanding of these stress responses, combining cross-layer evidence that connects gene regulation, protein activity, and metabolic remodeling. Despite these advancements, there are still challenges, particularly in the interpretation of complex datasets, the integration of multiple omic layers and the translation of results to real agricultural conditions. Finally, the review also discusses biotechnological approaches that may improve rice tolerance to pesticide exposure. In summary, the role of omic approaches to elucidate pesticide toxicity in rice and to contribute to more resilient crop production systems is critically reviewed.

## 1. Introduction

Rice (*Oryza sativa* L.) is a key resource for global food security, and is the main dietary resource for more than half of the world’s population [1]. Its nutritional constituents (carbohydrates, essential proteins, vitamins, minerals, antioxidant compounds) are indispensable in the prevention of malnutrition and in reducing the prevalence of chronic disease in developing regions [2]. However, production of rice is increasingly affected by climate changes, pest outbreaks, water scarcity, soil degradation, and post-harvest losses. All these factors threaten to compromise sustainability and food security in the most vulnerable societies [3,4].

In rice cultivation, pesticides are widely used to stabilize production and reduce the yield losses caused by weeds, insects, and pathogens [5]. Despite their agronomic benefits, continuous or intensive application of pesticides can compromise rice physiology and long-term productivity. Herbicides, fungicides, and insecticides disrupt metabolic homeostasis, enhance oxidative stress, impair photosynthetic efficiency and nutrient uptake, and interfere with hormone-regulated pathways, leading to reduced growth and yield [6].

Beyond the crop itself, pesticide residues in rice grains, irrigation water, and surrounding environments are multiple exposure routes for humans [7,8,9]. Chronic exposure has been associated with endocrine disruption, neurotoxicity, carcinogenic effects, and reproductive impairment, particularly in rice-dependent regions and among agricultural workers [10,11]. Monitoring data from 2016 to 2020 from China’s three main rice-growing regions [12] showed that 1.2%–4.0% of brown rice samples exceeded maximum residue limits (MRLs), with higher exceedance rates in the Yangtze River basin (2.88%) compared to the Northeast Plain (0.17%). Flooded paddy systems are prone to pesticide persistence in soils, sediments, and adjacent aquatic habitats due to anaerobic conditions and hydrological connectivity [13,14]. This behavior can disturb microbial communities and nutrient cycles, while also affecting non-target organisms by bioaccumulation and broader ecotoxicological effects [15].

These concerns align with the objectives of the UN 2030 Agenda for Sustainable Development, which calls for agricultural systems that increase productivity while improving environmental sustainability and resilience to biotic and abiotic stressors [1]. Although pesticides provide short-term protection that supports yield stability, their long-term use affects physiological crop performance together with ecological balance and public-health. Consequently, intensive pesticide use represents a major challenge for sustaining long-term productivity, environmental integrity, and human health in rice-based agroecosystems [9,11].

**Figure 1:**
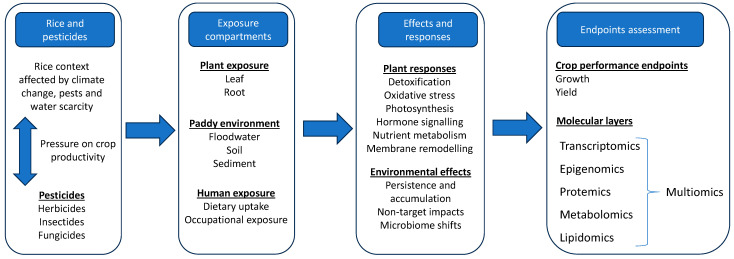
Conceptual overview of the experimental design, from the rice–pesticide system through exposure compartments and observed responses to endpoint assessment.

Conventional toxicological and physiological assessments in rice rely on phenotypic endpoints and therefore capture only a limited range of pesticide effects, as depicted in Figure 1. However, early molecular perturbations often arise before visible symptoms or measurable yield reduction occur, leaving early stress responses undetected [16]. Therefore, more advanced molecular approaches are required to achieve a holistic characterization of pesticide-induced stress in rice. In this context, high-throughput omic technologies, including transcriptomics, epigenomics, proteomics, and metabolomics, enable the systematic detection and interpretation of changes across multiple biological layers [17]. More specifically, transcriptomics and metabolomics have shown to be highly sensitive in detecting early biochemical and regulatory disruptions following pesticide exposure [18,19].

Compared to single-parameter assays, omic platforms provide clear advantages for analyzing complex stress responses in rice. These approaches capture coordinated molecular changes across genes, proteins, and metabolites (including lipids), rather than focusing on isolated markers [20]. Together, these platforms reveal interconnected mechanisms such as detoxification, redox regulation, epigenetic modulation, and membrane remodeling. When multiple omic layers are integrated within the same experimental framework, pesticide-induced stress responses can be interpreted as interconnected biological networks instead of independent molecular events [17]. This systems-level perspective improves mechanistic understanding and cross-study comparability.

Therefore, the main objective of this review is to critically synthesize and evaluate omic-based studies that characterize pesticide-induced stress responses in rice. The review focuses on the main omic layers that are responsible for transcriptional regulation, epigenetic modulation, protein-level responses, and metabolic reprogramming under pesticide exposure. Key challenges related to data interpretation and cross-study comparability are addressed, and the added value of multi-omic integration for rice-specific pesticide stress assessment is discussed. In summary, the review highlights both the strength and the current limitations of omic approaches in the context of pesticide toxicity in rise.

## 2. Pesticide Use in Rice Crops

In rice cultivation, pesticides remain a key tool to control insects, weeds, and diseases, but at the same time their use can trigger stress responses in the crop. Globally, pesticide compounds applied in rice cultivation are mainly classified as insecticides, herbicides, and fungicides, including both long-established active ingredients and more recently introduced molecules [21]. For insect control, organophosphates and carbamates have been used for decades, while pyrethroids and neonicotinoids (such as imidacloprid) have become more widespread in recent times, especially against insect pests such as leafhoppers and planthoppers. Classical insecticides, such as chlorpyrifos, are still extensively used and in some situations reported at higher doses than recommended, increasing the risk of phytotoxicity effects and residue accumulation [22,23]. Regarding herbicides, both pre- and post-emergence active ingredients are still prominent in the context of weed control in flooded rice fields [24]. Butachlor continues to be relevant in many areas, but the use of other herbicides such as penoxsulam has increased in the last decade due to their efficacy and persistence in paddy water [23,25]. In the case of fungicides, triazoles, strobilurins and benzimidazoles are commonly applied for diseases such as blast or sheath blight [26]. Tricyclazole is strongly related to blast control, whereas carbendazim is often found in agricultural surroundings due to its persistence, which is a source of concern for residue accumulation and environmental impact [27,28].

From a physiological viewpoint, pesticide exposure can interfere with central metabolic processes in rice. A common response is a reduction in photosynthetic capacity together with a lower chlorophyll content and slower growth, in line with the inhibition of protein synthesis and disturbance of energy balance [29]. At the biochemical level, oxidative stress represents a recurrent mechanism of injury [30]. Many pesticides can induce production of reactive oxygen species (ROS), triggering oxidative damage that is manifested by lipid peroxidation (usually measured as malondialdehyde, MDA), a marker of membrane impairment. In rice treated with pesticides such as chlorpyrifos, increased MDA levels have been reported together with compensatory activation of antioxidant enzymes (superoxide dismutase, SOD, and peroxidases), consistent with enhanced oxidative pressure [31]. This pattern reflects the dual nature of pesticides: while intended to protect the crop from pests and diseases, they can also simultaneously compromise essential physiological processes. In addition, phytotoxicity is not only manifested by oxidative damage but also involves broader metabolic reprogramming. Taken together, these alterations suggest that rice metabolism is biased towards protective responses (antioxidants, osmolytes, detoxification) at the expense of growth functions under conditions of high or sustained exposure [32].

Pesticide stress can interact with abiotic stressors and increase damage. For example, imidacloprid exposure under saline conditions can be more harmful than each factor alone due to increased oxidative stress and impaired detoxification, causing an increased internal pesticide load [33]. This result strengthens the argument that under realistic agricultural conditions, pesticide effects are not simply additive and can be amplified by concurrent environmental stressors [34].

Under chemical pressure, rice activates basal defense mechanisms that combine detoxification, antioxidant responses, and hormonal regulation [35]. Plant detoxification is described as a phased process: phase I reactions, mediated by cytochrome P450 enzymes, expose the xenobiotic to functional groups; phase II reactions, often involving glutathione S-transferases (GSTs) and glutathione, promote conjugation to increase solubility and reduce reactivity; and phase III involves transport and sequestration through proteins such as ATP-binding cassette (ABC) transporters, which compartmentalize or export the transformed compounds. This coordinated metabolic architecture is the basis of varietal differences in tolerance or sensitivity to certain pesticides [36]. Concurrently, antioxidant defenses (SOD, catalase, peroxidase, and other enzymes, together with non-enzymatic antioxidants such as glutathione or ascorbate) act to limit ROS damage and restore redox balance. At the signaling level, stress-related hormones involved (e.g., salicylic acid (SA), jasmonic acid (JA), ethylene and abscisic acid (ABA)) modulate the expression of detoxification and antioxidant genes and coordinate adaptive responses [37]. Finally, the accumulation of osmoprotectants such as proline, together with lipid remodeling, suggests that rice does not merely detoxify the pesticide, but undergoes broader physiological reprogramming to maintain membrane integrity, osmotic balance, and protein stability under chemical stress [38].

In summary, these basal defense mechanisms account for much of the response plasticity and provide the functional framework for interpreting the omic results discussed in the following sections.

## 3. The Omic Framework: From Genotype to Phenotype

**Figure 2:**
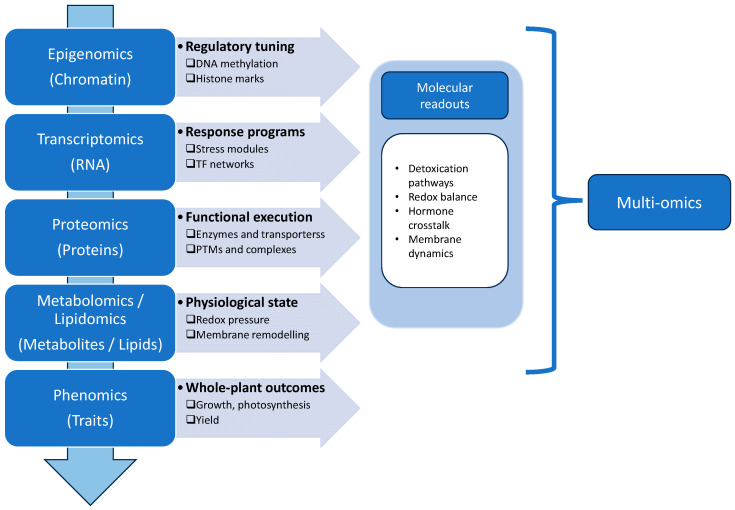
Schematic representation of the multi-omic framework linking epigenomic, transcriptomic, proteomic, metabolomic/lipidomic, and phenomic layers to integrated molecular readouts and whole-plant outcomes.

Deciphering pesticide-induced stress demands more than describing visible injury or measuring a limited set of biomarkers, due to the multifaceted and context-dependent nature of plant responses. A single exposure can activate detoxification programs, disrupt redox balance, alter membrane integrity and hormone signaling, and reallocate resources away from growth [39,40]. The value of the omic framework lies in its ability to organize biological information across successive layers along the genotype-to-phenotype axis (Figure 2). This view is particularly relevant for pesticide stress, where responses typically involve coordinated regulatory, biochemical, and physiological changes rather than disruption of a single pathway [41].

At the most fundamental level, genomics defines gene content and structural variation that influence detoxification enzyme families, transport systems, antioxidant capacity, and hormone signaling components [42]. Genomic approaches can help explain contrasting sensitivities among varieties exposed to the same pesticide conditions and identify loci associated with tolerance through enhanced detoxification, reduced uptake or translocation, or improved stress buffering [43]. However, genomic information alone rarely predicts real-world outcomes, as pesticide responses are strongly influenced by dose, timing, developmental stage, and interacting environmental stresses.

Epigenomics adds a regulatory layer by explaining how transcriptional reprogramming can occur without changes in DNA sequence. Chemical modifications of DNA and chromatin dynamically modulate gene accessibility, allowing rapid adjustment of stress-responsive pathways. In this way, epigenetic mechanisms allow selective and context-dependent use of the static genome in response to pesticide exposure [44,45].

Transcriptomics captures the immediate regulatory response to pesticide exposure, allowing the identification of coordinated gene modules related to detoxification, redox defense, membrane dynamics, and hormone-mediated signaling [46]. This layer differentiates broad stress responses, often linked to growth penalties, from more targeted detoxification and recovery programs associated with tolerance. Nevertheless, mRNA abundance does not necessarily reflect functional outcomes, as translation efficiency, protein turnover, and post-translational regulation can substantially modify the final biological response [47]. This distinction is especially relevant under pesticide stress, as key processes such as ROS scavenging, conjugation, and transport depend not only on mRNA levels, but on the abundance and activation state of functional enzymes [48].

Proteomics represents the functional layer and addresses this limitation by reporting the proteins actually deployed for detoxification, redox control, signaling, and structural remodeling [49]. It reveals whether transcriptional changes translate into functional enzyme pools and reveals regulatory processes that remain invisible at the mRNA level. In addition, post-translational modifications (phosphorylation, oxidation, ubiquitination, etc.) can rapidly modify protein activity and stability under pesticide stress [50,51]. Therefore, proteomics helps differentiate between gene induction and effective responses (functional protein changes), and may be useful to highlight bottlenecks when transcript accumulation does not result in corresponding protein responses [52]. Within integrated frameworks, proteomics acts as the interface between regulatory signals and the biochemical outcomes.

Downstream, metabolomics and lipidomics provide a direct window of the physiological state by integrating upstream gene regulation and protein activity under prevailing conditions. Metabolomics reflects the status of central carbon, nitrogen, and energy metabolism, while lipidomics resolves membrane remodeling through changes in lipid composition and saturation, both of which are critical under oxidative and chemical stress [53,54]. Together, these layers clarify resource trade-offs between efficient detoxification, metabolic disruption, and growth limitation [55].

Finally, phenomics links molecular alterations to whole-plant performance by quantifying growth, photosynthetic efficiency, biomass allocation, and stress symptoms across genotypes and environmental conditions [56,57]. This layer anchors omic interpretation by determining whether molecular responses translate into functional resilience or instead reveal hidden sensitivity.

Accordingly, the following sections focus on the omic layers that have provided the most robust mechanistic evidence for pesticide-induced stress in rice. Specifically, transcriptomics and epigenomics will be discussed as regulatory layers, proteomics as the functional interface, and metabolomics (including lipidomics) as direct biochemical readouts linking molecular regulation to stress phenotypes. This framework establishes a stepwise connection between regulatory shifts, molecular effectors, and metabolic outcomes that ultimately shape the stress phenotype.

## 4. Single-Omic Insights into Pesticide Responses in Rice

A comprehensive interpretation of pesticide effects on rice requires information from multiple molecular layers. Single-omic approaches provide insights into specific regulatory or biochemical levels, whereas multi-omic strategies allow coordinated interpretation across domains. This section reviews epigenomics, transcriptomics, proteomics, and metabolomics for the description of pesticide-induced molecular alterations in *O. sativa*, with a particular focus on rice-specific responses to pesticide exposure.

### 4.1. Epigenomics

Epigenomic mechanisms contribute to transcriptional plasticity in rice and are involved in plant responses to chemical stressors such as pesticides [58]. DNA methylation and histone modifications modulate chromatin accessibility and influence gene expression under herbicide treatment. In rice, DNA methylation occurs in CG, CHG, and CHH sequence contexts and is maintained or removed by specific DNA methyltransferases and demethylases, respectively. These processes support both genome stability and stress-responsive transcriptional control, as shown by Deng and Li [44,58].

Genome-wide methylome analyses show that herbicide exposure induces locus-specific changes in DNA methylation. For instance, atrazine treatment has been associated with altered methylation patterns at promoters, transposable elements, and stress-related genes correlating with transcriptional regulation [59]. These epigenomic changes affect genes involved in xenobiotic metabolism, hormone signaling, and oxidative stress pathways. This evidence indicates that DNA methylation acts as an active regulatory component of pesticide stress rather than a passive byproduct of stress. Similar epigenetic responses have been reported for other herbicides. In rice exposed to atrazine and acetochlor, targeted DNA demethylation was associated with enhanced herbicide degradation capacity, supporting a functional role of methylation dynamics in pesticide detoxification [60,61].

Histone modifications also contribute to chromatin-level regulation under pesticide exposure. In atrazine-treated rice, shifts in histone modification states have been associated with transcriptional activation of detoxification-related genes. In particular, enrichment of activating marks and reduction of repressive marks at herbicide-responsive loci [62]. Although these observations support a role for histone-mediated regulation, genome-wide histone profiling under pesticide stress remains scarce in rice, with most studies focused on specific genes or pathways.

Despite these advances, the interpretation of epigenomic responses to pesticide exposure remains challenging. DNA methylation and histone modifications are highly tissue- and developmental stage-specific, whereas most studies rely on bulk leaf or whole-plant material. Moreover, distinguishing causal epigenetic regulation from downstream stress-induced effects is often difficult. Variability in pesticide type, exposure design, and epigenomic methodologies further limits cross-study comparability. Consequently, the stability, reversibility, and potential heritability of pesticide-induced epigenetic modifications in rice are still poorly understood and require more integrative and longitudinal research [58,63].

### 4.2. Transcriptomics

Transcriptomic analyses have become a central approach to characterize molecular responses of rice to pesticide exposure. RNA sequencing enables genome-wide detection of differentially expressed genes across time- and dose-dependent treatments and allows identification of regulatory pathways that respond before visible symptoms or yield reductions are detected [64]. Pesticide exposure induces coordinated transcriptional programs, notably involving detoxification, redox regulation, transport, and stress signaling pathways [65].

A consistent feature of rice transcriptomes under pesticide stress is the regulation of genes associated with xenobiotic detoxification [19,65]. Phase I oxidative metabolism is reflected by the induction of cytochrome P450 monooxygenases, an expanded gene family in rice and provide broad substrate specificity. Transcriptomic profiling under herbicide exposure (quinclorac and isoproturon) has identified multiple CYP genes as responsive and their association with in primary pesticide oxidation [19,66]. These transcriptional changes are often accompanied by modulation of phase II conjugation pathways, with repeated upregulation of genes encoding glutathione S-transferases and UDP-glycosyltransferases indicating enhanced conjugation capacity and detoxification potential in rice tissues [67]. Phase III transport processes are also evident at the transcript level, as ATP-binding cassette transporters are frequently induced, which supports increased sequestration or export of conjugated metabolites after herbicide exposure [68].

Beyond detoxification enzymes, transcriptomic studies in rice also show modulation of antioxidant- and stress-related genes after exposure [65]. In parallel, hormone-related pathways, including auxin, abscisic acid, ethylene, and brassinosteroid signaling, are transcriptionally regulated under pesticide exposure [69,70]. These regulatory networks coordinate detoxification, stress adaptation, and growth responses in rice [71].

Stress-responsive transcription factors, such as members of the WRKY, NAC, bZIP, MYB, and AP2/ERF families, act as central regulators of these transcriptional programs [46]. Noncoding RNAs have also been reported as additional regulatory components, with contributions that vary depending on tissue type and exposure duration, adding temporal and spatial complexity to pesticide-induced gene regulation [72]. Similarly, transcriptomic analyses have implicated basic leucine zipper (bZIP) transcription factors in herbicide responses [73]. However, in rice, functional and transcriptomic validation of bZIP88 or its homologues under pesticide exposure is still lacking and requires rice-specific investigation.

Several studies have provided functional validation of candidate detoxification genes identified in transcriptomic analyses of rice exposed to isoproturon [74,75]. Genome-wide RNA-seq analysis showed that brassinosteroid signaling controls herbicide detoxification through the transcription factor OsBZR4. Loss of OsBZR4 function resulted in higher isoproturon accumulation and altered expression of degradation-related genes, whereas wild-type plants showed enhanced detoxification capacity. Metabolic profiling using UPLC–Q-TOF–MS/MS identified five phase I metabolites (formed via demethylation and hydroxylation) and five phase II conjugates (glycosides and amino acid conjugates) in rice grains, with concentrations reduced in bzr4-mutant lines compared to wild-type lines. Likewise, identification of the cytochrome P450 gene CYP76C6 at the transcript level led to its functional characterization using overexpression and CRISPR/Cas9-knockout approaches. Increased CYP76C6 expression reduced isoproturon accumulation and cellular damage, whereas knockout lines displayed higher herbicide retention, accompanied by altered metabolite and conjugate profiles. These results support that transcriptomic signatures can reflect active detoxification processes in rice.

Together, these results define a conserved, but rice-validated transcriptional framework for pesticide responses [19,70]. This framework involves early hormone- and kinase-mediated signaling, induction of phase I oxidation enzymes, activation of phase II conjugation pathways, reinforcement of antioxidant defenses, and increased transporter expression to facilitate sequestration and elimination of xenobiotics. Although the magnitude of these responses depends on pesticide chemistry, cultivar, tissue, and exposure conditions, the underlying regulatory architecture is reproducibly observed across a variety of rice transcriptomic studies.

Despite its sensitivity, transcriptomics alone does not provide direct information on protein abundance, enzyme activity, or metabolic flux. Therefore, transcriptional induction of detoxification genes following exposure cannot be assumed to translate into effective pesticide degradation without complementary evidence. In addition, transcriptional responses in rice are highly dependent on the experimental and biological context. Variations in cultivar, developmental stage, pesticide formulation, exposure duration, and interacting environmental stresses can substantially alter gene expression patterns, which complicates cross-study comparisons [39]. The presence of large and redundant gene families, particularly cytochrome P450s and conjugating enzymes, further hinder functional attribution based solely on transcript abundance [76].

Moreover, a fraction of differentially expressed genes may reflect secondary stress effects rather than direct pesticide action. For this reason, transcriptomics should be considered a hypothesis-generating layer that defines candidate regulatory and enzymatic components requiring further validation.

### 4.3. Proteomics

Proteomic studies provide direct insight into the functional impact of pesticide exposure in rice. While transcriptomic reflects regulatory changes, proteomics captures protein-level alterations more directly linked to physiological processes, including enzyme abundance, redox regulation, and metabolic activity. In rice, most pesticide-related proteomic studies have relied on two-dimensional gel electrophoresis coupled with MALDI–TOF mass spectrometry, whereas fully quantitative LC–MS/MS approaches remain less frequently applied [77,78].

A common proteomic response to pesticide exposure in rice is the modulation of detoxification- and defense-related proteins. In glyphosate-treated leaves, increased abundance of glutathione S-transferases (GSTs), thioredoxin h-type proteins, nucleoside diphosphate kinase, and peroxiredoxins has been reported. These changes are consistent with the activation of conjugation and redox-buffering systems [77]. Similarly, probenazole treatment induced GSTU17 together with enzymes of the phenylpropanoid pathway (e.g., phenylalanine ammonia-lyase (PAL) and caffeic acid O-methyltransferase (COMT)), indicating activation of defense-related secondary metabolism under chemical exposure [78].

Oxidative imbalance is a recurrent feature in rice proteomic response to pesticide exposure. For example, in quinclorac-treated plants, proteomic profiling revealed alterations in glutathione metabolism and chlorophyll biosynthesis. Salicylic acid pre-treatment enhanced the abundance of glutathione reductases (OsGR2, OsGR3) and aldehyde dehydrogenases (OsALDH2B5, OsALDH7), which was associated with improved detoxification and reduced metabolic disruption [79]. In addition, glyphosate exposure similarly induced antioxidant enzymes (e.g., ascorbate peroxidase, superoxide dismutase, peroxiredoxins, and GSTs), which clearly indicates the activation of redox defense systems [77]. Comparable antioxidant-dominated proteomic profiles were reported under paraquat exposure, a herbicide with strong redox activity, indicating that oxidative stress constitutes a common downstream effect of chemically distinct pesticides. Finally, in transgenic rice, quantitative proteomics further supported paraquat tolerance to enhanced antioxidant and polyamine-related pathways, with increased abundance of enzymes involved in putrescine and spermidine metabolism following overexpression of β-ketoacyl-CoA synthase (EiKCS) [80].

Proteomic analyses also indicate that pesticide exposure affects photosynthesis and primary metabolism in rice. A pronounced reduction in the ribulose-1,5-bisphosphate carboxylase/oxygenase (Rubisco) large subunit abundance was consistently observed in rice leaves treated with glyphosate or paraquat. This response linked pesticide stress to impaired carbon assimilation and altered energy balance [77]. Similar decreases in photosynthesis-related proteins have been reported in rice exposed to other agrochemicals, suggesting that suppression of primary metabolism is a common secondary effect of pesticide stress [78]. Reduced photosynthetic capacity may, in turn, limit the energy available for detoxification and repair processes and thereby intensifying physiological stress.

Despite the central role of cytochrome P450 monooxygenases and ATP-binding cassette (ABC) transporters in xenobiotic detoxification, direct proteomic evidence for their regulation in rice remains limited. Most support for these pathways derives from transcriptomic studies, transgenic data, or comparative analyses in other plant species, rather than from direct protein-level detection in rice tissues [79,81]. This gap highlights the need for more sensitive and targeted proteomic analyses in rice.

Distinct pesticides also generate compound-specific proteomic signatures. Probenazole primarily induces proteins associated with defense signaling and secondary metabolism, which is consistent with its function as a resistance-inducing compound rather than a classical herbicide [78]. In contrast, paraquat triggers accumulation of redox- and polyamine-associated proteins, reflecting its specific mode of action as a ROS generator. Proteomic studies on insecticide exposure in rice are less frequent, and their interpretation can be confounded by genetic background effects, as observed in comparisons between Bt-transgenic and wild-type rice lines under insecticide treatment [82].

Despite the mechanistic insight provided by proteomics, several limitations remain. Most studies rely on bulk tissues and single sampling time points, which mask spatial and temporal dynamics of protein regulation. In addition, traditional gel-based workflows have limited sensitivity for low-abundance proteins, membrane transporters, and post-translational modifications [77,78]. Functional validation of proteomic candidates is still limited, which constrains causal interpretation.

Overall, current evidence indicates that pesticide exposure in rice triggers coordinated proteomic responses characterized by enhanced antioxidant and detoxification capacity, reduced photosynthetic activity, and compound-specific metabolic adjustments. Integrating proteomic data with other omic layers will be necessary to clarify how these protein-level changes contribute to pesticide tolerance or susceptibility in rice.

### 4.4. Metabolomics

Pesticide exposure also triggers metabolomic changes in rice [18]. By quantifying low-molecular-weight compounds, metabolomic approaches are able to capture alterations in central metabolism, redox balance, and stress-related pathways that are not always predictable from transcript or protein levels alone [83]. Complementarily, lipidomics provides quantitative insight into membrane phospholipids, neutral lipids, sphingolipids, and lipid-derived signaling molecules, thereby resolving membrane remodeling processes under chemical stress [84]. In rice, both targeted and untargeted metabolomic (and lipidomic) strategies based on GC–MS and LC–HRMS have been applied to characterize pesticide-induced metabolic disturbances [85,86]. However, while metabolomics is relatively well represented, comprehensive mass spectrometry–based lipidomic studies in rice remain comparatively limited, and much of the available evidence on lipid changes derives from classical biochemical or physiological analyses.

Metabolomic analyses report strong changes in primary metabolism following herbicide or insecticide exposure (i.e., shifts in carbohydrates, amino acid and organic acid profiles) [87,88]. In parallel, changes in amino acid composition (increased proline and branched-chain amino acids) suggested activation of stress-related nitrogen metabolism [89].

Organic acid metabolism in also affected, as shown by metabolomic studies, particularly pathways associated with central energy metabolism. Perturbations in tricarboxylic acid cycle intermediates, including citrate, malate, and succinate, have been reported under herbicide stress. In rice exposed to diclofop-methyl, increased citrate accumulation was linked to altered citrate-metabolizing enzymes and enhanced citrate exudation, indicating targeted metabolic reprogramming of organic acid metabolism rather than nonspecific toxicity [90]. Additional metabolomic studies further show that chemical stress induces broader shifts in energy-related metabolic pathways, including glyoxylate and dicarboxylate metabolism, which supports coordinated reorganization of central carbon metabolism [6]. Together, these results indicate that pesticide-induced metabolic stress involves coordinated modulation of organic acid pools and energy-associated pathways.

Secondary metabolic pathways, including defense-related compounds, are also reprogrammed in pesticide-exposed rice plants [88]. Metabolomic profiling of chlorpyrifos-treated plants revealed marked perturbations in polyphenols and flavonoids, with many flavonoid compounds decreasing under high exposure levels. These changes reflect modulation of phenolic and antioxidant pathways that contribute to cellular defense and antioxidant capacity [89]. In related studies of rice stress responses, alterations in phenylpropanoid and flavonoid biosynthetic pathways have been consistently identified as key components of stress-associated metabolomic reprogramming, highlighting their central role in adaptive defense mechanisms [91].Together, these observations suggest that pesticide exposure reconfigures specialized metabolic networks linked to defense signaling and redox balance.

Several studies also report changes in glutathione, ascorbate, and other redox-related metabolites following pesticide exposure. Increased glutathione levels and shifts in the reduced-to-oxidized glutathione ratio have been observed in rice leaves treated with chlorpyrifos and other herbicides, which is consistent with activation of detoxification and antioxidant pathways. Alterations in phytohormone-associated metabolites (e.g., jasmonate-, salicylate-, and abscisic acid-related compounds) further indicate that pesticide stress reshapes metabolic routes involved in signaling and defense coordination [92].

Comparative metabolomic analyses across different herbicides reveal compound-specific metabolic fingerprints, reflecting differences in chemical structure, uptake, and biotransformation. In atrazine-treated rice, HRMS identified multiple Phase I metabolites and Phase II conjugates, indicating metabolic conversion of the parent compound [93]. In other cases, metabolomic analysis showed accumulation of intermediate transformation products that could be associated with enhanced toxicity. For example, exposure to phenamacril led to accumulation of intermediate metabolites predicted to be more toxic than the original fungicide, although further biotransformation reduced its persistence [94]. Several studies show that pesticide-induced metabolic disturbances in rice are dynamic and can be modulated by external factors. Hormonal regulation is one such mechanism. In rice seedlings exposed to s-metolachlor, application of gibberellin-3 partially restored altered metabolite profiles. Multi-omic analyses in rice exposed to S-metolachlor revealed increased flavonoid accumulation and enhanced peroxidase activity, which were associated with mitigation of oxidative damage and phytotoxicity [95].

Also, pesticide formulation and delivery strategies influence metabolic responses. In thifluzamide-treated seedlings, non-targeted metabolomics revealed disruptions in amino acid metabolism, protein biosynthesis, and secondary metabolism [96]. When delivered via mesoporous silica nanoparticles, these disturbances were attenuated. The nanoparticle formulation promoted amino acid and nucleotide metabolism, increased total protein content, and partial recovery of chlorophyll, phenol, and flavonoid levels. Similar formulation-dependent effects were observed in rice seeds treated with nano-formulated prothioconazole. Comparable formulation-dependent effects were observed in rice seeds treated with nano-formulated prothioconazole, which mitigated metabolic stress relative to conventional formulations despite higher uptake of the active ingredient [97].

Together, these results show that metabolic responses to pesticides in rice are not uniform and can be reshaped by hormonal and formulation factors. Metabolomics therefore provides a useful framework to distinguish primary toxic effects from modulated stress responses.

Lipidomics provides insight into membrane phospholipids, neutral lipids, and lipid-derived signaling molecules under stress. In rice, early studies showed that herbicide treatment affects sterol composition and membrane-associated enzyme activity [98], while oxidative damage to membrane lipids (evidenced by elevated malondialdehyde levels) represents a common downstream consequence across herbicide classes [99,100]. More recent studies have identified specific lipid alterations. Chlorpyrifos exposure significantly increased unsaturated fatty acids (e.g., FA 20:5, 4.50-fold), together with elevated phosphatidylcholine, phosphatidylethanolamine, and phosphatidylinositol levels [89]. Similarly, tricyclazole shifted fatty acid profiles toward greater unsaturation, with six lipid metabolism pathways (53.9% of affected pathways) showing differential regulation [6].

These coordinated changes are consistent with membrane remodeling processes aimed at maintaining fluidity, redox balance and lipid-mediated signaling capacity under chemical stress. Although methodological frameworks for integrated metabolome–lipidome analysis have been established [101], comprehensive LC–MS-based lipidomic profiling in rice remains limited. Evidence from other cereals indicates that phospholipid- and sphingolipid-mediated signaling contributes to abiotic stress adaptation [102,103], suggesting that similar lipid-mediated regulatory mechanisms may operate in rice under pesticide exposure.

Despite its analytical power, metabolomic assessment of pesticide responses in rice presents important limitations. The metabolome is highly dynamic and strongly influenced by developmental stage, tissue type, and environmental conditions, which again complicates cross-study comparability. Coverage remains incomplete, particularly for low-abundance or transient metabolites, and structural annotation of unknown features remains challenging. Moreover, metabolite levels reflect integrated outputs of transcriptional and enzymatic processes, limiting causal interpretation when metabolomics is applied in isolation. Lipid levels, although relevant for membrane remodeling and stress signaling, remains comparatively understudied in rice pesticide research. Limitations include insufficient resolution of lipid classes, lack of spatial information, and limited integration with transcriptomic and proteomic data [89,99].

Together, these metabolomic and lipidomic limitations highlight the need for high-resolution and integrative approaches to clarify the functional role of metabolic and lipid remodeling in pesticide responses.

## 5. Systems Biology: Multi-Omic Integration

As shown in the preceding sections, pesticide exposure in rice triggers responses that propagates across regulatory, biochemical and physiological scales. While single-omic approaches are valuable for identifying candidate genes or metabolites, multi-omic integration (combining at least two non-redundant omic layers such as transcriptomics with metabolomics, or proteomics with lipidomics) allows movement from descriptive lists of candidates towards mechanistic explanations [104]. This integrative approach is particularly important for studying pesticide-induced stress in plants, where phenotypes rarely result from disruption of a single pathway but rather from the interplay between damage and defense processes. By linking upstream regulation with downstream biochemical and physiological outcomes, multi-omic approaches help distinguish adaptive responses from secondary toxic effects [105]. Over recent years, multi-omic studies focusing on pesticide impacts in rice have converged on a limited set of recurring biological themes emerging from such integrative analyses.

First, pesticide-induced stress entails a metabolic reprogramming that is both pesticide-specific and context-dependent. Integration transcriptomics and metabolomics demonstrated that structurally distinct pesticides (butachlor, chlorpyrifos and tricyclazole) trigger divergent regulatory programs in rice [6]. The herbicide butachlor primarily perturbed carbohydrate metabolism, the insecticide chlorpyrifos reshaped amino acid pathways, and the fungicide tricyclazole altered fatty acid composition and lipid-related gene expression. These pathway-specific shifts were linked to differential transcriptional control of carbon, nitrogen, and lipid metabolism. This example illustrates how multi-omic integration connects regulatory and metabolic layers, revealing that pesticide responses are not uniform but compound-specific. Overall, such approaches provide richer mechanistic insight into how different pesticide classes reprogram rice metabolism.

A second domain where multi-omic integration is particularly informative is the assessment of redox imbalance and its relationship with detoxification and antioxidant systems. Pesticide exposure frequently disrupts redox homeostasis, and integrative analyses can capture coordinated responses across transcriptional, proteomic, and metabolic layers. For example, induction of detoxification genes, parallel shifts in enzyme abundance, and modulation of glutathione-associated metabolites. In rice treated with quinclorac, combined RNA-seq and iTRAQ proteomics revealed chlorophyll degradation and accumulation of redox-related intermediates, consistent with disturbed cellular homeostasis [79]. Salicylic acid pre-treatment modulated these responses by enhancing detoxification-related enzymes, as confirmed by transcript–protein correlation analyses. The convergence of signals across omic layers strengthened mechanistic interpretation and clarified how defense modulation mitigated herbicide-induced stress.

Multi-omic integration also enables a more comprehensive assessment of hormone signaling and stress crosstalk caused by rice exposure to pesticides. While single-omic studies often identify activation of regulatory programs associated with biotic or abiotic stress. However, multi-omic approaches can add a further dimension by enabling the relationship between timing and internal rhythms and pesticide responses to be explored. For example, Chen et al. [106] conducted a three-level study (epigenomics, transcriptomics and metabolomics) to examine how the rice circadian system interacts with herbicide responses. Although the different omic layers were handled largely independently rather than being fully integrated in the analysis, which is one of the limitations of many multi-omic examples in pesticide-treated rice, the results were conceptually linked to herbicide responses in the context of the rice circadian clock.

In studies where metabolomics and/or lipidomics constitute one of the integrated layers, membranes remodeling becomes an important aspect of interpretation. Because oxidative stress tends to affect membranes, many pesticide responses involve restructuring of lipid composition. A representative example is the study described above by Liu and Zhu combining transcriptomics and metabolomics [6]. Tricyclazole-induced stress was associated with upregulation of genes encoding oil-body membrane proteins and a shift in fatty-acid profiles from saturated towards unsaturated species, consistent with typical stress-related adjustments in membrane fluidity and lipid-mediated signaling processes. These results are particularly relevant in rice, where membrane stability and transport processes are critical for adaptation to flooded paddy environments, and where formulations or adjuvants may additionally influence membrane integrity.

**Figure 3:**
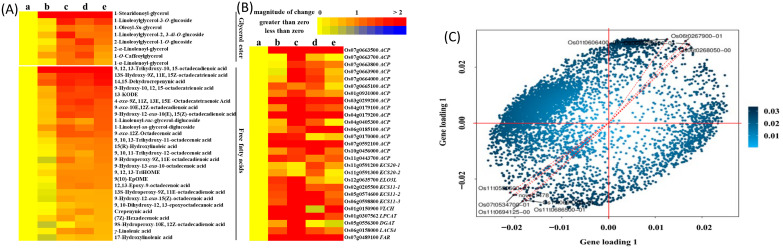
Integrated (**A**) metabolomic and (**B**) transcriptomic analyses showing ZNC-induced changes in fatty acid–related gene expression and lipid composition in DR tissues, with (**C**) O2PLS identifying Ko04626 pathway genes as major drivers of metabolomic variation. Adapted from Chongchong Lu et al. [107].

Multi-omics can also clarify trade-offs affecting yield and crop quality, which is essential for translating molecular insights into agronomic decisions. For example, Lu et al. [107] integrated transcriptomic and metabolomic analyses to compare biopesticide-based regimes with conventional chemical treatments under bacterial leaf streak stress. The authors reported that the biopesticide/elicitor-based treatment more effectively restored carbohydrate and amino-acid profiles (in both leaves and grains) and was associated with improved yield-related indices compared with conventional chemical pesticide treatments. As illustrated in Figure 3, joint integrative analysis linked key differentially expressed genes with metabolomic variation, providing a systems-level explanation of how treatment-specific molecular responses were connected to agronomic outcomes (Figure 3C).

Another valuable multi-omic strategy is the integration of molecular layers with explicit chemical characterization of pesticide transformation products. In the ametryn study by Qiao [108], combined transcriptomic and metabolomic were combined with chemical identification of metabolites and conjugates in rice tissues. This design enabled a step-by-step catabolic pathway to be described (including Phase I and Phase II reactions) and linked gene and metabolite changes to the appearance of pesticide-derived conjugates. Such integrative approaches are particularly powerful because they anchor biological interpretation to confirmed chemical species, reducing uncertainty about whether observed metabolic shifts reflect active detoxification, generalized stress metabolism, or both.

From an analytical perspective, multi-omic integration in studies of pesticide-exposed rice typically relies on three main strategies: (a) pathway enrichment analyses performed in parallel across layers, (b) correlation-based integration to connect differentially expressed genes/proteins with differentially accumulated metabolites, and (c) network- or module-based approaches that identify co-regulated multi-omic signatures. Across pesticide studies, these strategies frequently converge recurring functional modules, including detoxification pathways (e.g., P450s, GSTs, transporters), redox buffering systems, chlorophyll metabolism, amino acid reprogramming, and lipid remodeling. This convergence supports a systems-level interpretation that is more robust than analyzing individual layers in isolation. More broadly, the rice literature increasingly positions these integrative omic approaches as a route to building systems models of complex traits and stress responses [109].

However, several limitations still constrain the direct translation of multi-omic findings into field-level decisions [110]. One major challenge is temporal mismatch: transcriptomic responses often peak rapidly after exposure, whereas proteomic and metabolomic changes may appear later or reflect integrated responses over longer time windows. For this reason, time-series designs are essential to reconstructed plausible causal trajectories, from early signaling events to detoxification induction and metabolic stabilization. Another limitation concerns annotation depth, especially for metabolites and lipids, where many features remain unidentified or only ambiguously annotated. This limits the mechanistic interpretation even when cross-layer associations are strong. Cross-study comparability is further complicated by differences in analytical platforms, extraction protocols and normalization pipelines, which can alter the observable metabolome or proteome [111]. Finally, many experiments rely on controlled exposure conditions that do not fully capture real agricultural complexity (mixtures, repeated low-dose exposures, variable temperature/water regimes, and simultaneous pathogen pressure), factors that can substantially reconfigure response programs [112].

Looking forward, several directions could strengthen multi-omic inferences of pesticide stress in rice. First, broader incorporation of lipidomics would improve resolution of membrane-level mechanisms, complementing current transcriptome–metabolome integration. Second, spatially resolved or tissue-specific omics (roots versus shoots versus developing grain) could reveal compartment-dependent detoxification and allocation strategies, particularly relevant for root uptake and translocation processes of many pesticides [113]. Third, integrating omics with quantitative phenotyping (e.g., photosynthetic performance, chlorophyll fluorescence, recovery dynamics) would better anchor molecular signatures to agronomic outcomes. Finally, incorporating microbiome-aware designs will become increasingly important, since pesticide regimes can alter the rhizosphere and indirectly affect plant metabolism.

Collectively, multi-omic studies describe pesticide stress in rice as a coordinated systems-level response rather than the consequence of disruption to any single pathway, with direct implications for biomarker discovery and agronomic assessment. Their value lies not simply in generating more data, but in providing improved mechanistic resolution to connect regulatory programs with biochemical states and to distinguish between adaptive acclimation and toxicity.

## 6. Implications and Practical Applications

### 6.1. From Omic Signatures to Functional Outcomes in Rice

**Figure 4:**
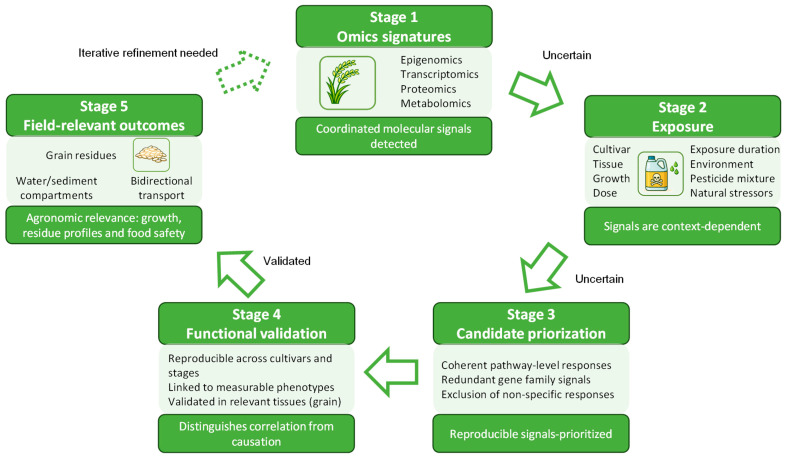
Translational framework linking omic signatures to functional outcomes in rice under pesticide exposure.

Multi-omic studies indicate that pesticide stress responses in *O. sativa* are coordinated across multiple biological layers, with recurring patterns involving Phase I–III detoxification, antioxidant defenses, and hormone-regulated pathways. These patterns are useful to identify candidate mechanisms. However, their translation into field-relevant tolerance should be treated as uncertain unless exposure conditions are comparable. Most experimental designs rely on single active ingredients, fixed doses, and controlled environments. In contrast, rice is cultivated under variable water management, fluctuating temperature and light, and repeated applications that can include mixtures and adjuvants. Under such scenarios, chemical and natural stressors can interact, potentially altering the direction or magnitude of molecular responses even for the same pesticide [34]. Figure 4 summarizes this context-dependent progression from multi-omic signal detection to functional interpretation. Early stages capture coordinated molecular changes that must be filtered according to biological and environmental context, distinguishing coherent pathway-level responses from redundant gene family activation or nonspecific stress effects. Functional validation, including genetic perturbation and assessment of reproducibility, physiological endpoints, and tissue relevance, represents the critical step separating correlation from causation. Overall, the framework emphasizes that omic signatures are hypothesis-generating and require rigorous, context-aware validation before agronomic relevance can be inferred.

This gap directly affects interpretation of so-called detoxification signatures. Induction of CYPs, GSTs, UGTs, and ABC transporters is often interpreted as evidence of improved detoxification capacity. However, transcript changes do not necessarily translate into enzyme activity or metabolic flux [48]. In addition, the presence of large and redundant gene families in rice complicates functional assignment based only on transcript abundance [114]. Therefore, a practical validation pipeline is essential. First, a candidate should show reproducible responses across cultivars and growth stages under comparable exposures. Second, the response should be linked to measurable endpoints, such as reduced pesticide accumulation, improved growth recovery, or lower oxidative damage. Third, residue-related endpoints should be evaluated in relevant tissues, especially grain.

Field-oriented interpretation should also consider environmental compartments specific to paddy systems. Pesticides can persist and migrate through flooded soils, and aquatic risk differs across rice-production strategies [115,116]. In addition, neonicotinoid dissipation and migration patterns have been quantified for paddy fields, and plant tissue transport has been described as bidirectional in rice. These dynamics affect exposure duration and residue profiles [117]. Therefore, omic markers should be interpreted together with exposure kinetics and compartment behavior rather than as stand-alone indicators.

Omic approaches can support precision breeding when molecular candidates are connected to causal function. In rice, this translational pathway has been illustrated for several herbicide responses. Brassinosteroid signaling was reported to control isoproturon detoxification through OsBZR4, with mutant lines showing higher herbicide accumulation and altered metabolite and conjugate formation [70]. Similarly, CYP90D5 was also reported to enhance degradation of isoproturon and acetochlor in both rice plants and grains, which links detoxification to an endpoint relevant for both phytotoxicity and residue outcomes [118]. CYP76C6 has been functionally validated through overexpression and CRISPR/Cas9-knockout approaches, where altered expression changed isoproturon accumulation and damage [75]. Together, these cases exemplify a translational continuum from transcriptomic discovery to functional validation and ultimately to trait-oriented development.

However, breeding for pesticide tolerance should rarely be approached as a single-gene solution. Detoxification capacity typically involves coordinated activity of multiple enzymes and transport processes. Regulatory nodes can also introduce trade-offs with growth and yield. Growth–defense balance is a well-established concept in plants, and similar trade-offs are plausible under pesticide stress [32]. Therefore, candidate targets should be prioritized when they are functionally validated, show limited penalties for growth and grain quality, and can be evaluated under realistic management.

Current precision breeding tools facilitate this process. The rice genome and its variation resources support candidate identification and marker-assisted strategies [42]. Gene editing has been discussed as a route to herbicide-resistant rice, although practical deployment depends on regulatory and risk assessment frameworks [43]. An effective strategy is to combine omics with high-throughput phenotyping platforms. Phenomics can reduce the phenotyping bottleneck, and rice-oriented platforms have been proposed to accelerate functional genomics and trait validation in rice [119]. Under this framework, omics can define candidate pathways, and phenomics can provide scalable validation under multiple exposures and environments.

### 6.2. Omic Evidence in Pesticide Management, Crop Safety, and Sustainability

Omic results can inform pesticide management by clarifying the dominant physiological disruptions associated with specific compound classes. Across studies, pesticide exposure in rice consistently induces oxidative imbalance, metabolic reprogramming, and hormone-mediated growth modulation. These effects can differ across pesticides and can be amplified by combined stresses. For example, imidacloprid was reported to exacerbate salt-stress effects in rice through chlorophyll-related and redox-associated mechanisms [33]. Such interactions support the need for management recommendations that account for co-occurring stresses rather than pesticide effects alone.

Crop safety should also be treated explicitly when discussing tolerance mechanisms. Seedling-based experiments may overlook processes that control residue deposition and grain composition. Alterations in grain quality under fungicide use have been reported, and tebuconazole application timing has been linked to metabolome shifts and quality-related traits in rice grain [87]. These observations indicate that enhanced tolerance does not automatically equate to preserved quality. Therefore, omic-guided tolerance strategies should be complemented by residue analysis and transformation-product profiling in edible tissues when the aim is translational impact.

Regulatory compliance is determined through residue frameworks rather than molecular stress markers. Codex maintains maximum residue limit (MRL) databases for rice commodities., with values that vary by pesticide and year of adoption [120]. In the EU, MRLs are defined under Regulation (EC) 396/2005, which establishes the legal framework for pesticide residues in food and feed [121,122]. These regulatory systems highlight an important practical consideration: mechanistic tolerance traits should be developed with residue compliance in mind, especially for traits that alter transport, sequestration, or grain allocation.

Sustainability arguments should be evidence-based. Pesticide use has been linked to environmental and food-safety concerns, and mitigation frameworks have been reviewed [5,21]. Rice paddy systems add specific risks due to the exposure of water and sediment compartments. Comparative assessments of aquatic risk under different rice-farming strategies further demonstrate that management choices directly influence environmental outcomes [115,116]. In this context, omics can support sustainability when applied to reduce reliance on repeated applications, identify lower-impact practices, or support integrated pest management decisions.

At present, omic endpoints are not yet formally incorporated into most regulatory frameworks. Their near-term value lies in providing mechanistic support for risk interpretation and early-stage screening rather than serving as stand-alone decision criteria. Important data limitations persist. Protein abundance does not necessarily correlate with transcript levels [48], and metabolomic or lipidomic data can also suffer from incomplete annotation and platform variability [123]. Accordingly, omic evidence should be framed as complementary. It can refine mechanistic hypotheses, support mode-of-action interpretation, and prioritize candidates for validation, but it should not be presented as a replacement for residue analytics, toxicological assessment, or field-based evaluation.

## 7. Conclusions and Future Perspectives

Multi-omic assessment has strengthened mechanistic understanding of pesticide stress in rice. Transcriptomics has repeatedly identified inducible detoxification and stress-response programs, and several rice studies have connected these regulatory modules to functional outcomes under herbicide exposure. Proteomics has provided additional evidence for pesticide-related oxidative stress and defense adjustments, although limited coverage and challenges in post-translational modification analysis still limit generalization. Metabolomics has revealed pesticide-specific metabolic reprogramming in rice, including shifts in amino acid pools, central energy metabolism, and secondary metabolites. In contrast, lipid-focused evidence remains less developed, despite the mechanistic relevance of membrane remodeling and lipid-mediated signaling in stress adaptation.

Important limitations continue to constrain translation. Many studies use bulk tissues and short exposure windows, which can obscure tissue specificity and temporal causality. Co-occurring environmental stresses may reshape the same molecular modules, complicating attribution to individual pesticide effects [124]. In addition, cross-layer correlations are common, whereas functional validation remains limited to a subset of candidates. OsBZR4 and CYP90D5 provide strong rice examples that link regulation to herbicide accumulation and metabolism, including grain-related endpoints [70,118]. However, many transcription-factor claims remain indirect, and non-rice evidence should be used only as supportive analogy.

Future research should prioritize field-relevant designs and stronger causal validation. Exposure scenarios need to incorporate mixtures, repeated low-dose applications, and realistic variability in water management and climate. Expanded time-series designs should be expanded to connect early regulatory events with later protein and metabolite states. Tissue-resolved approaches should also be developed, because transport and grain allocation are central for residue and food safety. Emerging single-cell and spatial multi-omic methodologies have been reviewed for plant sciences and could address these gaps when adapted to rice pesticide research [113]. In parallel, integrated studies that connect omics to yield and grain quality under complex stress contexts should be emphasized. A recent multi-omic study assessed bio- and chemical-pesticide effects on yield and quality under disease stress, which is closer to agronomic reality than single-factor assays [107].

From an applied perspective, the most defensible path is a tiered framework. Omics should serve to identify candidate mechanisms and biomarker panels, which must undergo validation through functional genetics, residue analytics, and multi-environment phenotyping. Rice breeding and genome-editing tools can accelerate this process, but deployment should remain consistent with regulatory requirements and sustainability goals [43]. Finally, pesticide-tolerance traits should always be evaluated alongside residue compliance, particularly in light of Codex and EU MRL frameworks that govern food safety decisions.

In summary, integrated multi-omics has improved mechanistic resolution for pesticide stress in rice. The next step should be stronger causal validation and more field-relevant designs. Under such conditions, omics can progress from descriptive signatures to practical strategies that safeguard production yield and grain quality while reducing environmental and human exposure risks in rice-based agroecosystems.

## Data Availability

No new data were created or analyzed in this study. Data sharing is not applicable to this article.

## Abbreviations

The following abbreviations are used in this manuscript.

ABA	abscisic acid
ABC	ATP-binding cassette
ALDH	aldehyde dehydrogenase
AP2/ERF	APETALA2/ethylene-responsive factor
bZIP	basic leucine zipper
CYP (P450)	cytochrome P450 monooxygenase
COMT	caffeic acid O-methyltransferase
DEGs	differentially expressed genes
DNA	deoxyribonucleic acid
FAO	Food and Agriculture Organization
GC-MS	gas chromatography–mass spectrometry
GST	glutathione S-transferase
HRMS	high-resolution mass spectrometry
iTRAQ	isobaric tags for relative and absolute quantification
JA	jasmonic acid
LC-HRMS	liquid chromatography–high-resolution mass spectrometry
LC-MS/MS	liquid chromatography–tandem mass spectrometry
MALDI-TOF	matrix-assisted laser desorption–ionization time-of-flight
MDA	malondialdehyde
MS	mass spectrometry
MYB	myeloblastosis transcription factor
NAC	NAM, ATAF, and CUC transcription factor family
*O. sativa*	*Oryza sativa*
O2PLS	two-way orthogonal partial least squares
OsBZR4	rice brassinosteroid signaling transcription factor 4
PAL	phenylalanine ammonia-lyase
PTM	post-translational modification
RNA	ribonucleic acid
RNA-seq	RNA sequencing
ROS	reactive oxygen species
Rubisco	ribulose-1,5-bisphosphate carboxylase/oxygenase
SA	salicylic acid
SOD	superoxide dismutase
TCA	tricarboxylic acid (cycle)
UN	United Nations
UGT	UDP-glycosyltransferase
WHO	World Health Organization
WRKY	WRKY transcription factor family
